# Predicting lymph node metastasis and prognosis of individual cancer patients based on miRNA-mediated RNA interactions

**DOI:** 10.1186/s12920-022-01231-x

**Published:** 2022-04-17

**Authors:** Shulei Ren, Wook Lee, Kyungsook Han

**Affiliations:** Department of Computer Engineering, Inha University, Incheon, 22212 South Korea

**Keywords:** Lymph node metastasis, miRNA–mediated RNA interaction, Prognosis, Competitive endogenous RNA

## Abstract

**Background:**

Lymph node metastasis is usually detected based on the images obtained from clinical examinations. Detecting lymph node metastasis from clinical examinations is a direct way of diagnosing metastasis, but the diagnosis is done after lymph node metastasis occurs.

**Results:**

We developed a new method for predicting lymph node metastasis based on differential correlations of miRNA-mediated RNA interactions in cancer. The types of RNAs considered in this study include mRNAs, lncRNAs, miRNAs, and pseudogenes. We constructed cancer patient-specific networks of miRNA mediated RNA interactions and identified key miRNA–RNA pairs from the network. A prediction model using differential correlations of the miRNA–RNA pairs of a patient as features showed a much higher performance than other methods which use gene expression data. The key miRNA–RNA pairs were also powerful in predicting prognosis of an individual patient in several types of cancer.

**Conclusions:**

Differential correlations of miRNA–RNA pairs identified from patient-specific networks of miRNA mediated RNA interactions are powerful in predicting lymph node metastasis in cancer patients. The key miRNA–RNA pairs were also powerful in predicting prognosis of an individual patient of solid cancer.

**Supplementary Information:**

The online version contains supplementary material available at 10.1186/s12920-022-01231-x.

## Background

The spread of cancer cells from the original (primary) tumor to another part of the body is called metastasis. During metastasis, cancer cells travel to other areas through either the bloodstream or the lymph system. As one of the steps of tumor metastasis, lymph node metastasis is commonly observed in cancer patients. Lymph node metastasis itself does not directly endanger the life of patients, but malignant tumors can metastasize to other parts of the body through lymph node metastasis [1]. Many studies have reported that the prognosis of patients with lymph node metastasis is worse than that of patients without lymph node metastasis [2]. Lymph node metastasis is also an important factor in determining effective treatment options for cancer patients.

Lymph node metastasis is usually detected based on the images obtained from clinical examinations. Recently deep learning methods such as convolutional neural networks (CNN) have been used to help clinicians detect lymph node metastasis in ultrasound images [3–5]. Detecting lymph node metastasis from ultrasound images is a direct and accurate way of diagnosing metastasis, but the diagnosis is often done after metastasis occurs.

Several studies have reported abnormal gene expression in the process of lymph node metastasis [6]. For example, the study of Okugawa et al. [7] suggested that the expression of KiSS1 is closely related to lymph node metastasis in colorectal cancer. Zhang et al. [8] predicted lymph node metastasis using differentially expressed mRNAs and noncoding RNAs. Dihge et al. predicted lymph node metastasis using gene expressions combined with clinicopathological characteristics [9].

Expression data of mRNAs and noncoding RNAs are valuable resources for studying and predicting lymph node metastasis. But, cancer is a complex and heterogeneous disease, so abnormal expression of individual genes cannot fully explain the development and metastasis of cancer. The development and metastasis of cancer is better explained by the dysregulation of gene interactions rather than by individual genes alone. For example, AKT1 is abnormally expressed in many types of cancer and the up-regulation of AKT1 has been known to be related to lymph node metastasis. But, recent studies found that miR-138 binding to AKT1 regulates the expression of AKT1 in tongue squamous cell carcinoma [10]. miR-519d inhibits lymph node metastasis by regulating MMP3 in oral squamous cell carcinoma and breast cancer [11, 12].

Salmena et al. [13] proposed a new gene regulation known as competitive endogenous RNA (ceRNA) hypothesis. The ceRNA hypothesis suggests that RNAs with similar miRNA response elements compete to bind to the same miRNA, thereby regulate each other indirectly. Motivated by the increasing evidence supporting the hypothesis, several computational methods have been developed to construct a network of ceRNAs [14, 15]. Most of the methods focused on mRNAs or lncRNAs only as ceRNAs and did not consider pseudogenes when constructing ceRNA networks.

In this study, we propose a new method for predicting lymph node metastasis based on differential correlations of miRNA-mediated RNA interactions in cancer. The types of RNAs considered in this study include mRNAs, lncRNAs, miRNAs, and pseudogenes. We constructed cancer patient-specific networks of miRNA mediated RNA interactions, and identified key miRNA–RNA interactions from the networks. We built a model using the correlations of the miRNA–RNA pairs as features for predicting lymph node metastasis. The model showed a much higher performance than other methods which use gene expressions alone. The key miRNA–RNA pairs were also powerful in predicting prognosis of individual patients in several types of cancer. The rest of this paper presents the method and the experimental results in several types of cancer.

## Results

### Prediction of lymph node metastasis

Using the $\Delta$PCCs of miRNA–RNA pairs obtained in our study, we predicted lymph node metastasis using the stacking model and base models (SVM and logistic regression) in seven types of cancer. As expected, the stacking model showed the better performance than the other models both in cross-validation and in independent testing (Additional file 1).

We compared the performance of stacking models using two different types of features: $\Delta$PCC of miRNA–RNA pairs and RNA expression. $\Delta$PCC of miRNA–RNA pairs was computed by equation 4 in the Methods section. For RNA expression feature, we used the RNAs with a *p*-value $<~0.01$ both in differential analysis between normal samples and tumor samples and in additional differential analysis between lymph node metastasis samples and non-metastatic samples. The performance of the stacking models was evaluated by fivefold cross-validation and independent testing using several measures: sensitivity, specificity, accuracy, positive predictive value (PPV), negative predictive value (NPV) and area under curve (AUC).

Tables 1 and 2 show the performance of two stacking models in the fivefold cross validation and in the independent testing, respectively. The stacking models with $\Delta$PCCs as features showed a better performance than those with RNA expressions both in the fivefold cross validation and in independent testing, except for thyroid cancer (THCA) in independent testing. These results indicate that $\Delta$PCC of miRNA–RNA pairs is a more powerful feature than the gene expression level in predicting lymph node metastasis, which in turn supports that lymph node metastasis is associated with dysregulation of gene interactions rather than individual genes, as mentioned in the Background section.

**Table 1:** Performance of the prediction model with different types of features in the fivefold cross validation

Cancer	Feature	#Features	#PCs	SN	SP	ACC	PPV	NPV	AUC
BRCA	EXP	5119	430	0.674	0.709	0.692	0.694	0.689	0.691
	$\Delta$PCC	1563	480	**0.773**	**0.806**	**0.790**	**0.796**	**0.784**	**0.789**
COAD	EXP	835	100	0.360	0.935	0.758	0.711	0.767	0.647
	$\Delta$PCC	1969	80	**0.760**	**0.965**	**0.902**	**0.905**	**0.901**	**0.862**
HNSC	EXP	292	10	0.750	0.684	0.720	0.739	0.696	0.717
	$\Delta$PCC	800	100	**0.956**	**0.877**	**0.920**	**0.903**	**0.943**	**0.917**
LUAD	EXP	6193	110	0.477	0.882	0.741	0.683	0.759	0.679
	$\Delta$PCC	12,981	200	**0.593**	**0.944**	**0.822**	**0.850**	**0.813**	**0.769**
LUSC	EXP	1371	190	0.644	0.867	0.786	0.736	0.809	0.756
	$\Delta$PCC	2436	200	**0.875**	**0.934**	**0.912**	**0.884**	**0.929**	**0.904**
STAD	EXP	476	120	0.905	0.472	0.763	0.778	0.708	0.688
	$\Delta$PCC	17,445	60	**0.973**	**0.903**	**0.950**	**0.953**	**0.942**	**0.938**
THCA	EXP	4205	30	0.663	0.663	0.663	0.634	0.691	0.663
	$\Delta$PCC	3397	150	**0.674**	**0.723**	**0.700**	**0.682**	**0.716**	**0.698**

In comparison of two types of features (RNA expression vs. deltaPCC), the better performances are shown in bold

In all cancer types, prediction with $\Delta$PCCs showed a better performance than that with RNA expression levels

PC, principal component; SN, sensitivity; SP, specificity; ACC, accuracy; PPV, positive predictive value; NPV, negative predictive value; AUC, area under the curve; EXP, RNA expression level

**Table 2:** Performance of the prediction model with different types of features in an independent testing

Cancer	Feature	#Features	#PCs	SN	SP	ACC	PPV	NPV	AUC
BRCA	EXP	5119	430	0.664	0.710	0.688	0.690	0.685	0.687
	$\Delta$PCC	1563	480	**0.776**	**0.826**	**0.802**	**0.813**	**0.792**	**0.801**
COAD	EXP	835	100	0.563	0.932	0.819	0.783	0.829	0.747
	$\Delta$PCC	1969	80	**0.906**	**0.986**	**0.962**	**0.967**	**0.960**	**0.946**
HNSC	EXP	292	10	0.867	0.792	0.833	0.839	0.826	0.829
	$\Delta$PCC	800	100	**0.967**	**0.792**	**0.889**	**0.853**	**0.950**	**0.879**
LUAD	EXP	6193	110	0.622	0.943	0.832	0.852	0.825	0.782
	$\Delta$PCC	12,981	200	**0.784**	**0.971**	**0.907**	**0.936**	**0.895**	**0.878**
LUSC	EXP	1371	190	0.533	0.808	0.707	0.615	0.750	0.671
	$\Delta$PCC	2436	200	**0.889**	**0.962**	**0.935**	**0.930**	**0.938**	**0.925**
STAD	EXP	476	120	0.937	0.452	0.777	0.776	0.778	0.694
	$\Delta$PCC	17,445	60	**0.905**	**0.968**	**0.926**	**0.983**	**0.833**	**0.936**
THCA	EXP	4205	30	**0.737**	0.796	0.768	0.757	**0.778**	**0.766**
	$\Delta$PCC	3397	150	0.658	**0.864**	**0.768**	**0.807**	0.745	0.761

In comparison of two types of features (RNA expression vs. deltaPCC), the better performances are shown in bold

In all cancer types except thyroid cancer (THCA), prediction with $\Delta$PCCs showed a better performance than that with RNA expression levels

PC, principal component; SN, sensitivity; SP, specificity; ACC, accuracy; PPV, positive predictive value; NPV, negative predictive value; AUC, area under the curve; EXP, RNA expression level

We also compared the performance of our method with that of Zhang’s method [8] using the same data sets and the same SVM model. Among the seven types of cancer used in our study, comparison was made for four types of cancer because the four cancer types are common to both studies. The train_score and test_score in Table 3 were obtained using the scikit-learn package, which was used by Zhang’s study. In all cancer types used in comparison, our model which used $\Delta$PCCs as features was better than the four SVM models of Zhang’s method, which used the expression levels of mRNAs, miRNAs and lncRNAs separately. These results also demonstrate that $\Delta$PCCs of miRNA–RNA pairs are much more powerful features than expression data of RNAs when predicting lymph node metastasis.

**Table 3:** Comparison of the performance of our SVM model with that of Zhang’s SVM model [8]

Cancer	Method_feature	Train_score	Test_score
BRCA	Our model_$\Delta$PCC	**0.972**	**0.787**
	Zhang_mRNA	0.798	0.680
	Zhang_miRNA	0.764	0.737
	Zhang_lncRNA	0.793	0.696
COAD	Our model_$\Delta$PCC	**0.984**	**0.905**
	Zhang_mRNA	0.849	0.871
	Zhang_miRNA	0.902	0.886
	Zhang_lncRNA	0.869	0.871
LUAD	Our model_$\Delta$PCC	**0.996**	**0.850**
	Zhang_mRNA	0.808	0.849
	Zhang_miRNA	0.885	0.795
	Zhang_lncRNA	0.798	0.849
LUSC	Our model_$\Delta$PCC	**0.999**	**0.904**
	Zhang_mRNA	0.871	0.900
	Zhang_miRNA	0.939	0.847
	Zhang_lncRNA	0.861	0.900

In comparison of two types of features (RNA expression vs. deltaPCC), the better performances are shown in bold

Among the seven types of cancer used in our study, comparison was made in four types of cancer because they are the only common cancer types in both studies. The train_score and test_score were obtained using the scikit-learn package, which was used by Zhang’s study. In all four caner types, our model showed the better performance in both training and testing. our model_$\Delta$PCC: SVM model using $\Delta$PCCs as features. Zhang_X: SVM model using the expression levels of RNA type X as features

### Overall survival of cancer patients

We analyzed the overall survival of cancer patients by performing a log-rank test with respect to $\Delta$PCCs of miRNA–RNA pairs obtained in this study. Table 4 shows top three miRNA–RNA pairs with the smallest *p*-value from the log-rank test in each type of cancer. The remaining miRNA–RNA pairs with *p*-value less than 0.01 are available in Additional file 2.

**Table 4:** Comparison of *p*-values from the log-rank test with miRNA–RNA pair, and individual RNA and miRNA involved in the pair

Cancer	miRNA–RNA pair	Type of RNA in the pair	*P*-value of miRNA–RNA pair	*P*-value of miRNA	*P*-value of RNA
BRCA	miR-26b_AC079414.1	lncRNA	2.270E−05	9.203E−01	5.896E−01
	miR-3192_PPDPFL	mRNA	6.320E−05	1.351E−03	1.346E−02
	miR-3192_AC013549.3	lncRNA	.260E−04	5.028E−01	1.346E−02
COAD	miR-604_AL162426.1	lncRNA	1.869E−04	4.365E−01	6.730E−01
	miR-3679_RPL26P29	Pseudogene	3.122E−04	1.315E−02	8.171E−01
	miR-6835_AC037459.2	lncRNA	7.746E−04	9.815E−01	2.938E−02
HNSC	miR-4539_KRTAP10-2	mRNA	1.849E−04	3.033E−01	1.629E−03
	miR-6730_LINC01435	lncRNA	9.783E−04	1.038E−02	3.211E−03
	miR-5195_AL390067.1	lncRNA	1.070E−03	8.716E−02	3.435E−02
LUAD	miR-581_LINC00628	lncRNA	4.719E−07	1.925E−02	8.736E−01
	miR-7848_AC087588.2	lncRNA	2.220E−06	1.750E−05	7.506E−01
	miR-3680-1_AL138789.1	lncRNA	1.300E−05	2.386E−02	5.371E−01
LUSC	miR-548z_PNLIPRP2	Pseudogene	1.175E−04	3.178E−01	6.640E−04
	miR-3972_CSAG4	Pseudogene	1.485E−04	5.168E−01	4.740E−01
	miR-146b_PHETA2	mRNA	1.488E−04	4.779E−02	2.760E−01
STAD	miR-604_OLFML3	mRNA	1.000E−05	4.787E−02	4.921E−01
	miR-554_OR10A5	mRNA	4.040E−05	4.727E−03	5.852E−02
	miR-149_OR10A5	mRNA	1.689E−04	4.727E−03	8.850E−01
THCA	miR-5685_GADD45A	mRNA	3.489E−03	7.915E−01	2.587E−01
	miR-6784_AC093281.2	lncRNA	3.762E−03	5.934E−01	5.559E−02
	miR-8071-2_CFB	mRNA	3.991E−03	1.392E−02	9.494E−01

As shown in Table 4, the *p*-values from the log-rank test with $\Delta$PCC are much smaller than those with individual RNAs involved in the miRNA–RNA pairs. Three pseudogenes (RPL26P29, PNLIPRP2, and CSAG4) are included in the top three miRNA–RNA pairs with the smallest *p*-value (Table 4), and several miRNA-pseudogene pairs were found as potential prognostic pairs for all 7 types of cancer (Additional file 2).

**Fig. 1:**
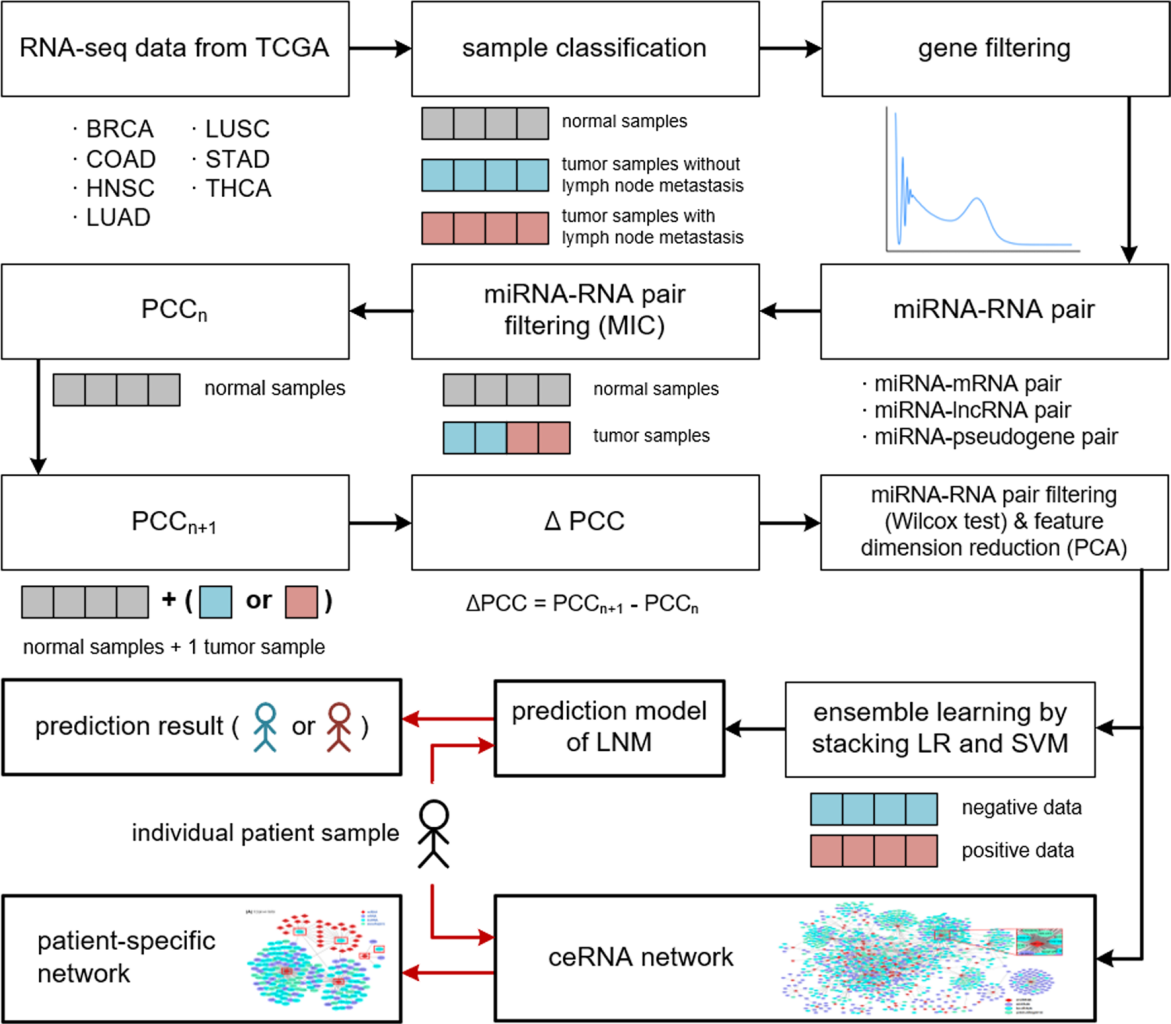
Overall survival rates of patients with respect to $\Delta$ **PCCs of miRNA–RNA pairs in 7 cancer types.** $\Delta$PCCs of miRNA–RNA pairs are predictive of the survival rates of patients in all 7 types of cancer

**Fig. 2:**
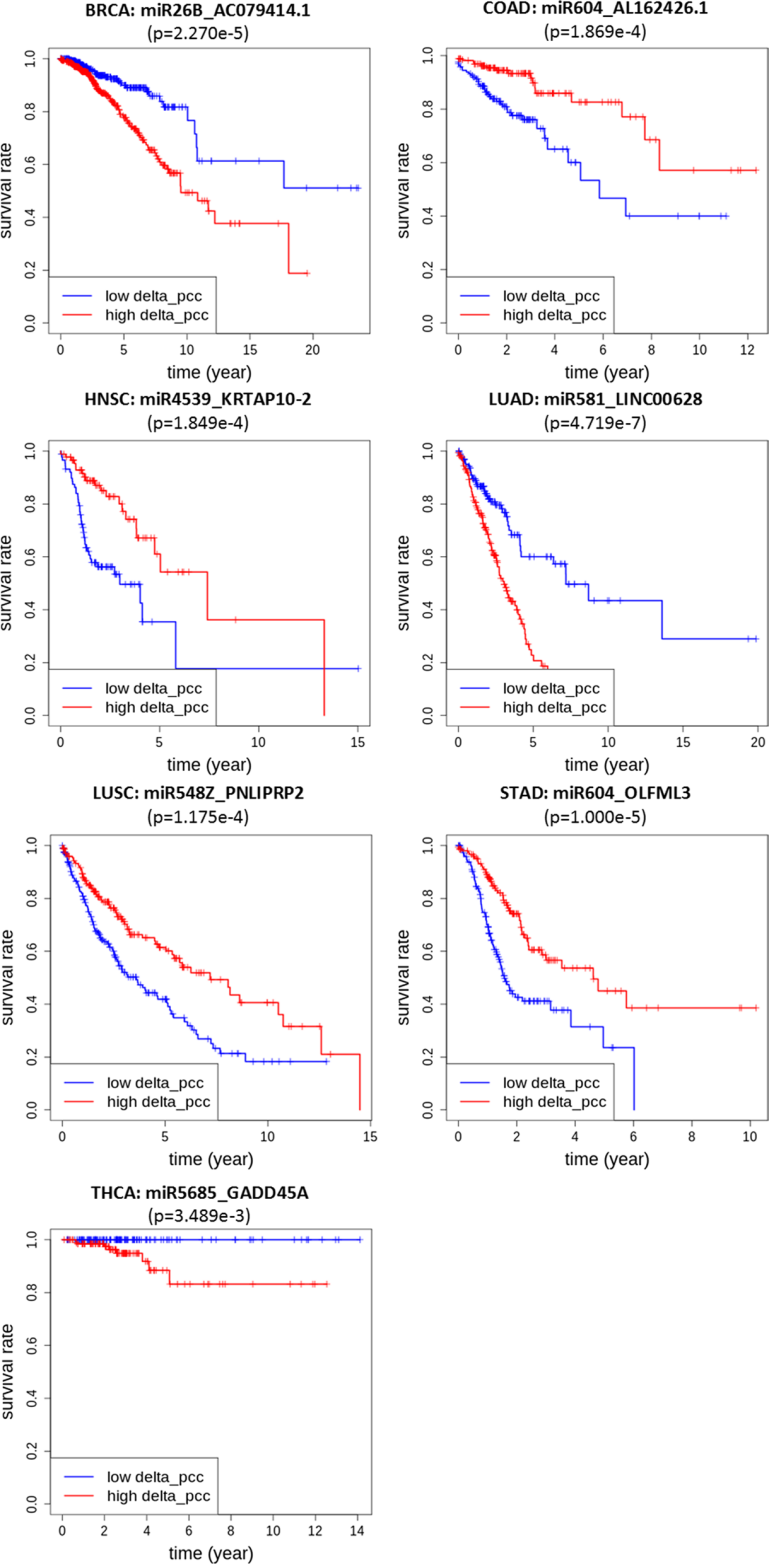
Overall survival rates of patients with respect to expressions of individual RNAs in Fig.  1. In contrast to the miRNA–RNA pairs, none of the individual RNAs showed predictive power of the survival rates of cancer patients

Figure 1 compares the overall survival rates of two groups of patients with respect to $\Delta$PCC of miRNA–RNA pairs in 7 types of cancer. In all 7 types of cancer, $\Delta$PCCs of miRNA–RNA pairs were powerful in predicting the survival rates of patients. For comparative purposes, Fig. 2 shows the overall survival rates of patients of BRCA, COAD and LUAD with respect to RNA expressions instead of $\Delta$PCC of miRNA–RNA pairs. The RNAs involved in the miRNA–RNA pairs of Fig. 1 (miR-26b_AC079414.1 pair for BRCA, miR-604_AL162426.1 pair for COAD, and miR-581_LINC00628 for LUAD) were selected for the comparison. None of the individual RNAs involved in the pairs showed predictive power of the survival rates of cancer patients, whereas the miRNA–RNA pairs were very powerful in predicting the survival rates of patients as demonstrated in Fig. 1.

### ceRNA networks

**Fig. 3:**
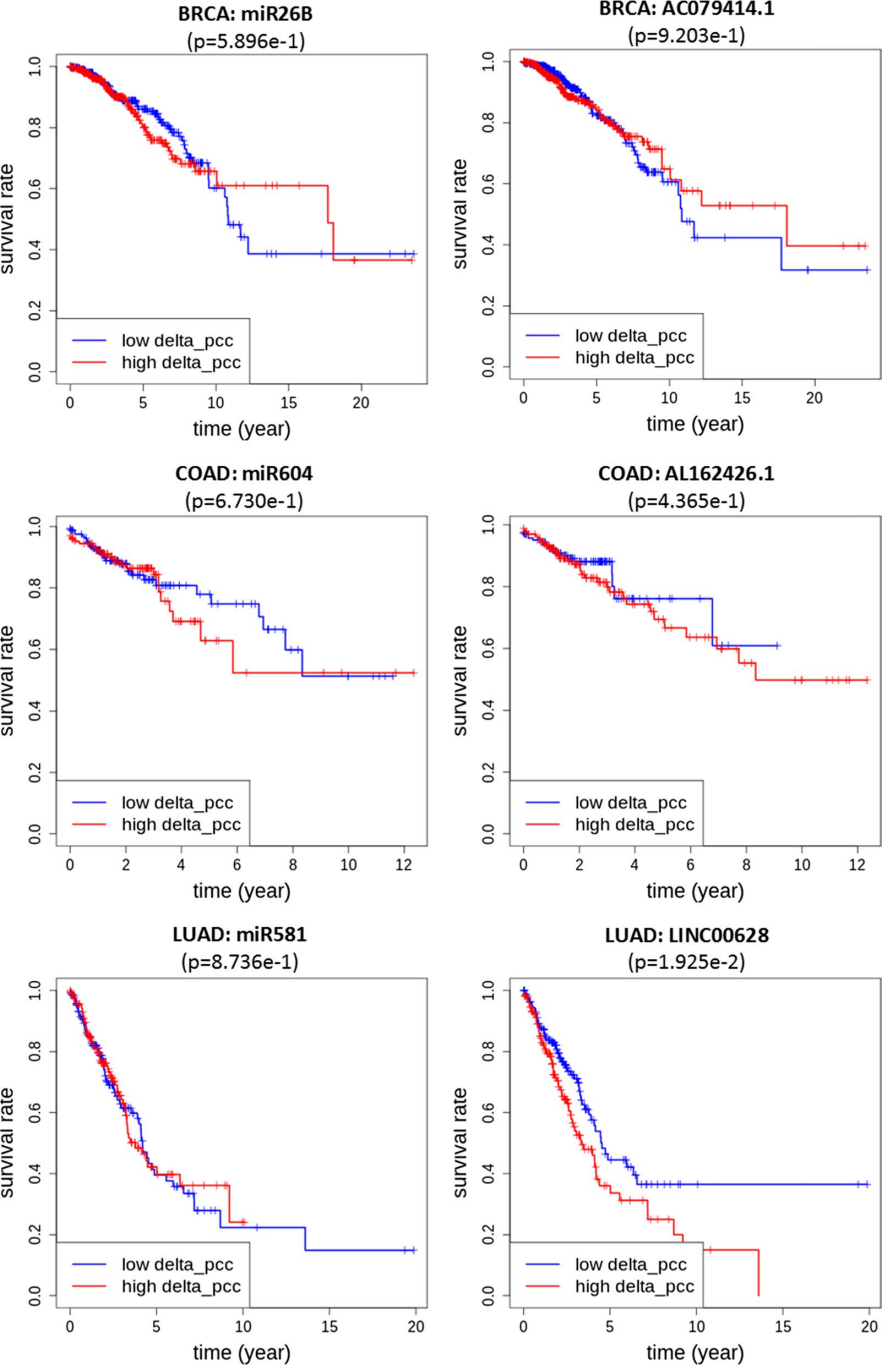
ceRNA network for breast invasive carcinoma (BRCA). The network is composed of 1563 miRNA–RNA interactions among 119 miRNAs, 423 lncRNAs, 380 mRNAs and 252 pseudogenes. The small network centered at miR-149 is a blowup of the subnetwork enclosed by a red box

For every tumor sample in Table 5, we constructed a ceRNA network and derived $\Delta$PCC of miRNA–RNA pairs from the network. We then constructed ceRNA networks with the miRNA–RNA pairs. Figure 3 shows a ceRNA network composed of all miRNA–RNA pairs for breast invasive carcinoma (BRCA). The network includes 1563 miRNA–RNA interactions among 119 miRNAs, 423 lncRNAs, 380 mRNAs and 252 pseudogenes. The small network centered at miR-149 is a blowup of the subnetwork enclosed by a red box.

**Table 5:** The number of normal samples, tumor samples, tumor samples with lymph node metastasis, and tumor samples without lymph node metastasis in seven types of cancer

Cancer	#Normal samples	#Tumor samples	#Lymph node metastasis	#Non-metastasis
BRCA	113	1102	447	457
COAD	41	478	107	242
HNSC	44	500	98	81
LUAD	59	533	123	231
LUSC	49	502	149	259
STAD	32	375	210	103
THCA	58	502	127	145

miR-149 is a miRNA that interacts with ceRNAs most frequently in the ceRNA network. miR-149 is known to promote metastasis in breast cancer when it is down regulated [16]. The ceRNA network also contains several genes associated with breast cancer. For instance, mutations in ERBB4 have been known to be associated with breast cancer [17]. Overexpression of YWHAE increases the proliferation, migration and invasion ability of breast cancer cells [18]. KAT6A promotes SMAD3 binding to oncogenic chromatin modifier TRIM24 and disrupts its interaction with the tumor suppressor TRIM33, which lead to the tumor cell metastasis in breast cancer [19].

**Fig. 4:**
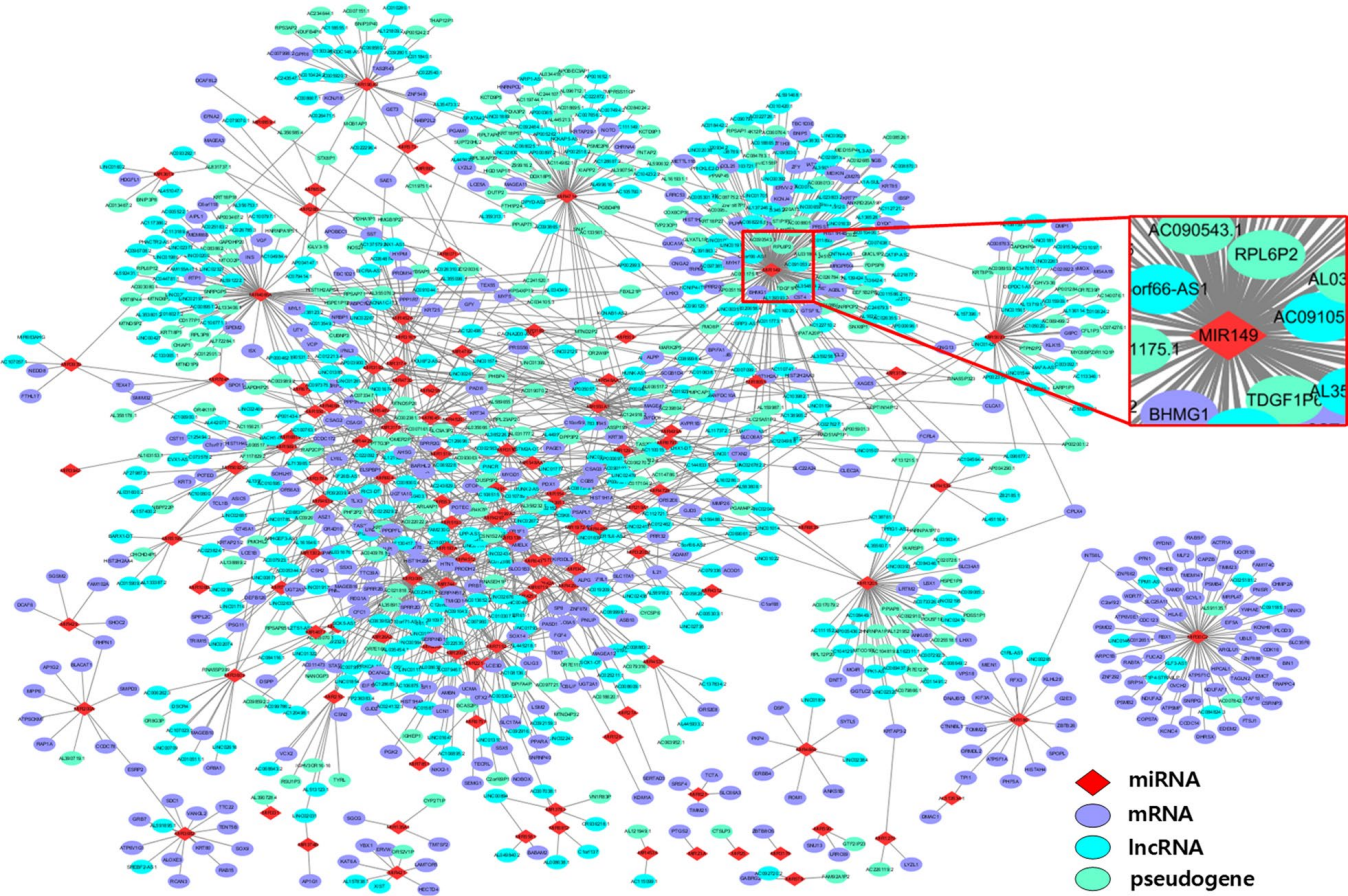
Subnetworks of patient-specific ceRNA networks for two LUAD patients. **A** LUAD patient (TCGA-44-7670) with a high $\Delta$PCC of the miR-581_LINC00628 pair. **B** LUAD patient (TCGA-NJ-A55O) with a low $\Delta$PCC of the miR-581_LINC00628 pair. The RNAs involved in the three miRNA–RNA pairs of Table 4 are marked by red boxes. For clarity, subnetworks of the three miRNA–RNA pairs are displayed

As an example of patient-specific networks, Fig. 4 shows the ceRNA networks specific to two LUAD patients with different $\Delta$PCCs of the miR-581_LINC00628 pair. Figure 4A is a ceRNA network for a LUAD patient (sample ID: TCGA-44-7670) with a high $\Delta$PCC group of the pair, whereas Fig. 4B is a ceRNA network for a LUAD patient (TCGA-NJ-A55O) with a low $\Delta$PCC group of the same pair. The network in Fig. 4A is composed of 210 miRNA–RNA pairs among 29 miRNAs, 77 lncRNAs, 47 mRNAs and 38 pseudogenes, and the network in Fig. 4B is composed of 111 miRNA–RNA pairs among 5 miRNAs, 53 lncRNAs, 30 mRNAs and 19 pseudogenes.

Apparently, the network in Fig. 4A includes more RNAs and interactions among them than that in Fig. 4B. As shown earlier in Fig. 1, patients with a high $\Delta$PCC of the miR-581_LINC00628 pair have a much lower survival rate than those with a low $\Delta$PCC of the pair. Similar observations were made in the other types of cancer.

## Discussion

The result of our work showed that $\Delta$PCCs of miRNA–RNA pairs derived from patient-specific ceRNA networks are more powerful than the expression levels of individual RNAs in predicting lymph node metastasis. This is related with dysregulated ceRNA interactions in cancer [20]. For instance, miR-125b may induce breast cancer metastasis by binding to STARD13 [21]. HOXD-AS1 prevents miR-130a-3p mediated degradation of SOX4 through competitive binding to miR-130a-3p, thereby promoting hepatocellular carcinoma transfer [22]. MT1JP regulates gastric cancer progression by binding to miR-92a-3p competitively with FBXW7 [23].

Unlike other studies on ceRNA interactions, our study considered pseudogenes as well as mRNAs and lncRNAs as ceRNAs. Pseudogenes were previously considered as genomic junk and neglected in the studies on ceRNA interactions as well. However, several experimental evidences suggested that pseudogenes can act as ceRNAs in the development of disease [24–26]. For instance, Karreth et al. [27] demonstrated that the pseudogene BRAFP1 functions as a ceRNA and induces lymphoma in vivo. Overexpression of the oncogenic pseudogene BRAFP1 promotes the formation of human B-cell lymphomas through serving as a ceRNA of the parental gene BRAF [28]. In prostate cancer, the pseudogene PTENP1 functions as a ceRNA to regulate PTEN expression by sponging miR-499-5p [29]. Straniero et al. [30] demonstrated that the pseudogene GBAP1 can function as a ceRNA for the glucocerebrosidase gene GBA by sponging miR-22-3p, thus revealing a new regulatory network in the pathogenesis of Parkinson’s Disease.

There are limitations in our current work. A patient-specific ceRNA network consists of miRNA–RNA pairs with significant changes from other patients by including miRNA–RNA pairs whose $|\Delta$PCC| is larger than the median $|\Delta$PCC| of all tumor samples of the same type. Since we used $|\Delta$PCC| instead of $\Delta$PCC, a patient-specific network does not show the direction of change (i.e., increase or decrease) in PCC. In the future, we plan to come up with a better way of presenting such information in a patient-specific network. Another direction of future work is to improve the performance of the prediction model further, in particular for thyroid carcinoma.

## Conclusion

The spread of tumors has always been a difficulty in tumor treatment, especially large-scale spread, which greatly reduces the survival rate of patients. Lymph node metastasis is the first step in the spread of many tumors. Therefore, predicting lymph node metastasis can make medical interventions in advance and reduce the risk of large-scale spread.

In this study, we constructed ceRNA networks for 7 types of solid cancer. Unlike other ceRNA networks, our ceRNA networks include pseudogenes as well as mRNA and lncRNAs. From the miRNA–RNA pairs in the ceRNA networks, we built a prediction model of lymph node metastasis in tumor samples.

Experimental results of the prediction model showed that $\Delta$PCCs of miRNA–RNA pairs from ceRNA networks are powerful for predicting lymph node metastasis in tumor samples. Comparison of our method with the features of other methods using the same data sets showed that $\Delta$PCCs of miRNA–RNA pairs are much more powerful than gene expression levels in predicting lymph node metastasis of cancer patients. Some miRNA–RNA pairs were also powerful in predicting prognosis of individual patients. Our work is preliminary and requires further investigation for clinical use. However, this approach will help characterize individual cancer patients and predict the occurrence of lymph node metastasis in advance.

## Methods

**Fig. 5:**
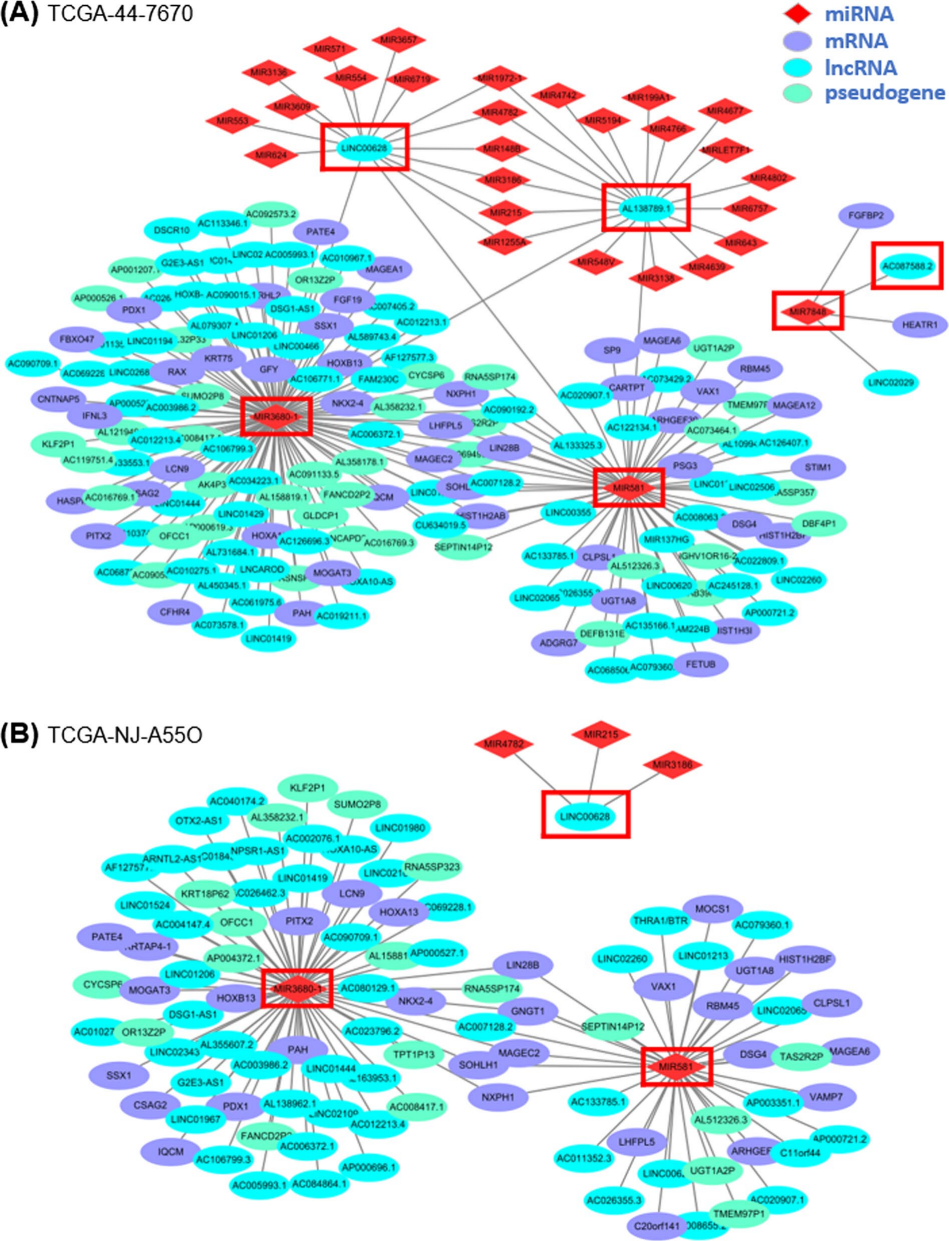
The overview of the overall workflow. There are three types of samples: normal samples (gray), tumor samples without lymph node metastasis (sky blue) and tumor samples with lymph node metastasis (pink). In our prediction model, tumor samples with lymph node metastasis (pink) and tumor samples without lymph node metastasis (sky blue) are treated as positive and negative instances, respectively

The overall workflow of our method is shown in Fig. 5. It shows data collection, data filtering, data processing, generation of miRNA–RNA gene pairs, and construction of a prediction model and patient-specific ceRNA network.

### Data collection

We collected gene expression profiles of lncRNAs, mRNAs, pseudogenes, and miRNAs and clinical profiles from The Cancer Genome Atlas (TCGA) data portal [31] for primary tumor samples of all solid cancer types. Normal samples of each type of cancer were also obtained from the TCGA data portal. All the gene expression profiles used in this study were obtained by RNA-sequencing (RNA-seq).

In TCGA, there were 18 types of solid cancer which have at least 200 samples. Among the 18 types, 6 types were excluded due to insufficient data on lymph node metastasis in their tumor samples. In the remaining 12 types of solid cancer, we selected the types which have at least 30 normal samples and 50 tumor samples with lymph node metastasis. Only 7 types of solid cancer satisfied such criteria: breast invasive carcinoma (BRCA), colon adenocarcinoma (COAD), head and neck squamous cell carcinoma (HNSC), lung adenocarcinoma (LUAD), lung squamous cell carcinoma (LUSC), stomach adenocarcinoma (STAD) and thyroid carcinoma (THCA).

The clinical profiles of the TCGA data includes the Tumor, Node, Metastasis (TNM) stage of samples. Samples with an M stage of 1 were excluded because distant organ metastasis often coexists with lymph node metastasis and makes the evaluation of prediction difficult. Based on the TNM staging system, we clustered the tumor samples into those with lymph node metastasis and and those without lymph node metastasis.

- Samples with lymph node metastasis: tumor samples with T stage of 1–4, N stage of 1–3, and M stage of 0
- Samples without lymph node metastasis: tumor samples with T stage of 1–4, N stage of 0, and M stage of 0

Table 5 shows the number of normal samples, tumor samples, tumor samples with lymph node metastasis, and tumor samples without lymph node metastasis in 7 types of cancer. The TCGA barcodes of all normal samples and tumor samples of Table 5 are provided as Additional file 3. The TCGA barcode is the primary identifier of biospecimen data in the TCGA project.

### Gene filtering

The gene names of the TCGA data are represented by Ensembl ID. Thus, we obtained the annotation files from the Ensembl project [32] and determined the names and biotypes of the genes (mRNAs, lncRNAs, pseudogenes and miRNAs). Table 6 shows the number of genes and their types.

**Table 6:** The number of RNAs of four biotypes in each cancer type studied in this study

Cancer	#miRNAs	#mRNAs	#lncRNAs	#pseudogenes
BRCA	165	18,084	8553	5528
COAD	157	17,573	7284	5304
HNSC	95	18,018	7427	4643
LUAD	197	18,054	8755	5954
LUSC	161	18,227	8706	5680
STAD	379	18,617	10,354	9039
THCA	153	17,568	7342	4753

We filtered out genes with an average count below 1. In RNA-seq data, counts are non-negative integer values. The count of unexpressed genes is 0, so the count of expressed genes is at least 1. Since the genes with the average count $< 1$ are unexpressed genes in most samples, we removed them. We then normalized the RNA-seq data of the genes using the trimmed mean of M values (TMM) method [33].

### Deriving miRNA–RNA pairs and feature selection

As mentioned earlier, any of lncRNAs, mRNAs, and pseudogenes with common miRNA response elements compete to bind to the same miRNA, so can act as competitive endogenous RNAs (ceRNAs). To obtain initial miRNA–RNA pairs we computed the maximal information coefficient (MIC) [34] of each miRNA with ceRNA candidates, which include mRNAs, lncRNAs, and pseudogenes. The overall workflow of our method for deriving miRNA–RNA pairs, selecting features and building a model can be summarized as follows:

1. Given RNA-seq gene expression data of miRNAs and ceRNAs (mRNAs, lncRNAs and pseudogenes), compute MIC of miRNA–RNA pairs in tumor samples and normal samples.
2. Select those miRNA–RNA pairs with MIC $\ge$ 0.5 in tumor samples or normal samples, and remove the remaining miRNA–RNA pairs.
3. Compute the Pearson correlation coefficient (PCC) of each miRNA–RNA pair in normal samples.
4. Recompute PCC in normal samples perturbed by a single tumor sample.
5. Compute the difference in PCC ($\Delta$PCC) between the normal samples and perturbed samples.
6. Select miRNA–RNA pairs with a *p*-value $<~0.01$ in the Wilcox test based on $\Delta$PCC, and remove the remaining pairs.
7. Reduce the dimension of feature vectors by the principal component analysis (PCA) of $\Delta$PCCs.

Our approach to predicting lymph node metastasis is based on the differential correlations of miRNA–RNA interactions of a sample from normal samples. To obtain the differential correlations of miRNA–RNA interactions of a sample, we first selected miRNA–RNA interactions with the maximal information coefficient (MIC). Pearson correlation coefficient (PCC) is the most commonly used for gene association. However, we used MIC instead of PCC to select potential miRNA–RNA pairs for a few reasons: (1) PCC can measure linear association only, but MIC measures linear or non-linear association between two variables. (2) MIC is less susceptible to outliers than PCC.

RNAs of the miRNA–RNA pairs are scattered into the two-dimensional space, which is divided into $n_X \times n_Y$ bins in the X and Y axes, Here X denotes the expression level of miRNA and Y denotes the expression level of any one of mRNA, lncRNA, or pseudogene in the pairs. Based on the number of scattered points in each bin, we calculate the mutual information *I*(*X*, *Y*) by Eq. (1). This process is repeated until the largest mutual information is obtained as the MIC (Eq. 2).

1 $$\begin{aligned} I(X,Y)= \sum _{X,Y}{p(X,Y)\log _2 \frac{p(X,Y)}{p(X)p(Y)}} \end{aligned}$$

where X: miRNA; Y: mRNA, lncRNA, or pseudogene

2 $$\begin{aligned} MIC(X,Y)= \max \limits _{n_X * n_Y<B}\frac{I(X, Y)}{\log _2 \min (n_X,n_Y)} \end{aligned}$$

The parameter B of MIC controls how much of the characteristic matrix we search over. Setting B too high can lead to non-zero scores even for random data, while setting B too low results in searching only for simple patterns [34]. we used the default setting for B, the 0.6th power of the number of samples, because the default setting is known to work well in practice [34].

Unlike the parameter B, there is no default setting for MIC. When selecting miRNA–RNA pairs for analysis, the threshold for MIC was set to 0.5, which is the median of its range [0, 1]. Setting the threshold of MIC smaller than 0.5 results in more miRNA–RNA pairs, which will contain a large number of spurious pairs. In contrast, with a larger threshold, we may miss potential prognostic gene pairs.

MICs of miRNA–RNA pairs were computed separately in tumor samples and normal samples because the association strength of miRNAs and ceRNAs are different between tumor and normal samples. Those miRNA–RNA pairs MIC $<~0.5$ in normal samples and tumor samples were removed because their association is not strong enough to be included in a ceRNA network.

We constructed a ceRNA network by subtracting a reference network for a group of normal samples from a perturbed network with a single tumor sample. Thus, each edge in the patient-specific network represents a differential PCC ($\Delta$PCC) of miRNA–RNA pair between a single tumor sample and a group of normal samples. MIC was not used at this stage because $\Delta$MIC does not make sense by its definition and $\Delta$PCC is more suitable for quantifying the perturbation by a single sample.

3 $$\begin{aligned} PCC(X, Y)= \frac{\sum _{i=1}^{n}{(X_{i}-{\bar{X}})(Y_{i}-{\bar{Y}})}}{\sqrt{\sum _{i=1}^{n}{(X_{i} -{\bar{X}})^2}\sum _{i=1}^{N}{(Y_{i}-{\bar{Y}})^2}}} \end{aligned}$$

where n: number of samples; X: miRNA; Y: mRNA, lncRNA, or pseudogene

4 $$\begin{aligned} \Delta PCC(X, Y) = PCC_{n+1}(X, Y) - PCC_{n}(X, Y) \end{aligned}$$

Every edge of a ceRNA network represents $\Delta$PCC of a miRNA–RNA pair, which is obtained by the following procedure:

1. Compute PCC of every miRNA–RNA pair in *n* normal samples.
2. Recompute PCC in $n+1$ samples which include *n* normal samples and a single tumor sample.
3. Compute differential PCCs ($\Delta$PCCs) between normal samples and the tumor sample.

We divided the $\Delta$PCCs of miRNA–RNA pairs into 2 groups, lymph node metastasis and non-metastasis, and performed the Wilcox test [35] in the two groups. We selected miRNA–RNA pairs with a *p*-value less than 0.01 in the Wilcox test. We reduced the number of miRNA–RNA pairs further by PCA. Table 7 shows the number of miRNA–RNA pairs left after each filtering process.

**Table 7:** The number of features left after each filtering process. miRNA–RNA pairs with MIC $<~0.5$ both in normal samples and tumor samples were removed by MIC filtering

Cancer	#Features after	#Features after	#Features after
	MIC filtering	Wilcox test	PCA
BRCA	90,837	1563	480
COAD	178,973	1969	80
HNSC	67,020	800	100
LUAD	341,146	12,981	200
LUSC	165,765	2436	200
STAD	976,763	17,445	60
THCA	38,077	3397	150

The miRNA–RNA pairs with a *p*-value $\ge$ 0.01 were removed by the Wilcox test. The number of features was further reduced after dimension reduction by PCA of $\Delta$PCCs. In both MIC filtering and the Wilcox test, each feature represents a miRNA–RNA pair, In PCA, the number of features indicates the dimension of a feature vector

### Construction of a prediction model

A model for predicting lymph node metastasis in tumor samples was built using an ensemble learning method. There are several ensemble learning methods such as bagging, boosting and stacking [36, 37]. Stacking is known to have higher prediction accuracy, yet lower risk of overfitting than bagging and boosting [38–40].

We selected support vector machine (SVM) and logistic regression (LR) as base models and combined them using stacking ensemble learning in the scikit-learn library [41]. We first trained the SVM model and LR model (base learners) with the original training set. We then used their prediction results as features to train a secondary learner. We used LR as the secondary classifier, which is the default classifier in the library. Stacking integrates the prediction results of the base learners in the best way through the secondary learner.

The tumor samples obtained from TCGA were divided into training and test sets with the ratio of 7:3. The parameters of the prediction model were determined by the grid search in the training set. When training and validating the prediction model, tumor samples with lymph node metastasis were considered as positive instances, and tumor samples without lymph node metastasis were considered as negative instances.

### Construction of a ceRNA network

For each type of cancer, we constructed a ceRNA network with the miRNA–RNA pairs obtained by the Wilcox test. A node of the ceRNA network represents one of miRNA, mRNA, lncRNA or pseudogene, and an edge represents the interaction of miRNA with other RNAs.

The patient-specific ceRNA network is a sub-network of the ceRNA network. For each miRNA–RNA pair, we computed the median of the absolute value of $\Delta$PCC (i.e., $|\Delta$PCC|) of the pair in all tumor samples of the same cancer type. A patient-specific ceRNA network was constructed by selecting the miRNA–RNA pairs whose $|\Delta$PCC| is larger than the median $|\Delta$PCC|. Thus, the edges in a patient-specific ceRNA network represent the miRNA–RNA interactions which show a significant change from other patients of the same cancer type.

## Supplementary Information

All additional files are available at http://bclab.inha.ac.kr/LNM/.

**Additional file 1**. Performance of two base models (logistic regression and SVM) and the ensemble model by stacking the base models in predicting lymph node metastasis.

**Additional file 2**. Potential prognostic miRNA–RNA pairs in seven types of cancer.

**Additional file 3**. TCGA barcodes of all normal samples and tumor samples studied in our work.

## Data Availability

The TCGA barcodes of all normal samples and tumor samples studied in our work are available in Additional file 3. The source code for generating miRNA–RNA pairs is available at https://github.com/rslrl/LNM.

## Abbreviations

CNN: Convolutional neural network
ceRNA: Competitive endogenous RNA
TCGA: The Cancer Genome Atlas
miRNA: MicroRNA
lncRNA: Long noncoding RNA
mRNA: Messenger RNA
MRE: miRNA response element
BRCA: Breast invasive carcinoma
COAD: Colon adenocarcinoma
HNSC: Head and neck squamous cell carcinoma
LUAD: Lung adenocarcinoma
LUSC: Lung squamous cell carcinoma
STAD: Stomach adenocarcinoma
THCA: Thyroid carcinoma
TNM: Tumor, Node, Metastasis
TMM: Trimmed mean of M values
PCC: Pearson correlation coefficient
MIC: Maximal information coefficient
SVM: Support vector machine
PPV: Positive predictive value
NPV: Negative predictive value
AUC: Area under curve
